# Biomarkers in psoriatic arthritis: A meta-analysis and systematic review

**DOI:** 10.3389/fimmu.2022.1054539

**Published:** 2022-11-30

**Authors:** Theo Wirth, Nathalie Balandraud, Laurent Boyer, Pierre Lafforgue, Thao Pham

**Affiliations:** ^1^ Rheumatology Department, Sainte Marguerite Hospital, Aix-Marseille University, APHM, Marseille, France; ^2^ Autoimmune Arthritis Laboratory, INSERM UMRs1097, Aix Marseille University, Marseille, France; ^3^ School of Medicine, EA 3279, CEReSS, Research Center on Health Services and Quality of Life, Aix Marseille University, Marseille, France

**Keywords:** psoriatic arthritis, psoriatic biomarker, psoriasis, arthritis, meta-analysis

## Abstract

**Introduction:**

Psoriatic arthritis (PsA) is a chronic inflammatory disease that frequently develops in patients with psoriasis (PsO) but can also occur spontaneously. As a result, PsA diagnosis and treatment is commonly delayed, or even missed outright due to the manifold of clinical presentations that patients often experience. This inevitably results in progressive articular damage to axial and peripheral joints and entheses. As such, patients with PsA frequently experience reduced expectancy and quality of life due to disability. More recently, research has aimed to improve PsA diagnosis and prognosis by identifying novel disease biomarkers.

**Methods:**

Here, we conducted a systematic review of the published literature on candidate biomarkers for PsA diagnosis and prognosis in MEDLINE(Pubmed), EMBase and the Cochrane library with the goal to identify clinically applicable PsA biomarkers. Meta-analyses were performed when a diagnostic bone and cartilage turnover biomarker was reported in 2 or moredifferent cohorts of PsA and control.

**Results:**

We identified 1444 publications and 124 studies met eligibility criteria. We highlighted bone and cartilage turnover biomarkers, genetic markers, and autoantibodies used for diagnostic purposes of PsA, as well as acute phase reactant markers and bone and cartilage turnover biomarkers for activity or prognostic severity purposes. Serum cartilage oligometrix metalloproteinase levels were significantly increased in the PsA sera compared to Healthy Control (HC) with a standardized mean difference (SMD) of 2.305 (95%CI 0.795-3.816, p=0.003) and compared to osteoarthritis (OA) with a SMD of 0.783 (95%CI 0.015-1.551, p=0.046). The pooled serum MMP-3 levels were significantly higher in PsA patients than in PsO patients with a SMD of 0.419 (95%CI 0.119-0.719; p=0.006), but no significant difference was highlighted when PsA were compared to HC. While we did not identify any new genetic biomarkers that would be useful in the diagnosis of PsA, recent data with autoantibodies appear to be promising in diagnosis, but no replication studies have been published.

**Conclusion:**

In summary, no specific diagnostic biomarkers for PsA were identified and further studies are needed to assess the performance of potential biomarkers that can distinguish PsA from OA and other chronic inflammatory diseases.

## 1 Introduction

Psoriatic arthritis (PsA) is a chronic inflammatory disease that develops in up to 30% of patients with psoriasis (PsO), and can affect up to 0.7% of the general population (1, 2). PsA is characterized as affecting axial and peripheral joints and entheses, which can present clinically with diverse symptoms, often resulting in delayed diagnosis and treatment. PsA can lead to progressive articular damage, thus can be a source of impaired function, permanent disability, quality of life, and an increase in mortality (3, 4).

Through the Biomarkers Project, the Group for Research and Assessment of Psoriasis and Psoriatic Arthritis (GRAPPA) places critical emphasis on the research of biomarkers in its development strategy (5). A biomarker is defined as a characteristic that is objectively measured and evaluated as an indicator of pharmacologic responses, or normal or pathogenic biological processes, for a therapeutic intervention (6). Identification of specific biomarkers would improve early diagnosis and management of PsA in patients with joint pain and/or skin psoriasis. Although PsA can develop in up to 30% of PsO patients, the prevalence of undiagnosed PsA in patients with psoriasis is still estimated to be 10-15% (4). Although classification criteria are sometimes used by default, there are currently no diagnostic criteria or specific biomarkers available for PsA (4). Therefore, we sought to identify biomarkers for determining diagnosis and prognosis of PsA by conducting a systematic review and pairwise meta-analysis.

## 2 Methods

To conduct this research, we followed the guidelines and the Preferred Reporting Items for Systematic Reviews and Meta-Analyses (PRISMA) statement for reporting on studies evaluating healthcare interventions (7). PRISMA Checklist is provided in supplementary file 1. Ethics approval was not required under local legislation for this study.

### 2.1 Study selection

A systematic search of English-language literature was conducted in MEDLINE (via PubMed), EMBase, and the Cochrane Library dating from inception to March 1, 2022. We designed the search algorithm according to the Patient-Intervention-Comparison-Outcome-Time (PICOT) format. Search terms corresponded to MeSH or Emtree terms for “psoriatic arthritis” and “biomarkers” or “pharmacological biomarkers”. A manual search was also performed.

After searching with the pre-determined PICOT algorithm, study eligibility was ascertained after reading the title, keywords, and abstract. Once the articles of interest were identified, the full text was read to evaluate it according to the exclusion criteria, and to subsequently extract the necessary data. Inclusion criteria required the following: study must be an observational or interventional clinical trial published in English before March 2022; include an assessment of biomarker(s) in serum (including genetic biomarkers), synovial fluid, urine, or feces as diagnostic or prognostic factors; cohort must include patients with PsA according to a rheumatologist diagnosis, Moll and Wright criteria, or Classification Criteria for Psoriatic Arthritis (CASPAR).

Exclusion criteria were applied in a sequential order, and included an editorial or congress abstract; duplicates (between electronic databases or journals); non-English language full text; non-human, non-PsA, or pediatric (≤18 years old) populations; off-topic; not relevant for diagnosis and prognosis in PsA. We focused on prognostic factors of disease severity, regardless of treatment, and did not include prognostic factors of response to treatment, out of the scope.

Study results were highlighted in the main text if select biomarkers were mentioned in at least 2 publications. In addition, all included articles are presented in tabular form (**Tables 1**, **2**).

### 2.2 Data extraction

All data was extracted into a standardized spreadsheet. For each article, we collected the data according to a pre-specified strategy. Collected information included the year of publication, name of the first author, geographical area, study design, population age and sex, disease duration, how the PsA population was determined (classification criteria used or therapist diagnosis), biomarkers investigated, primary study methodology, proportion of patients using corticosteroid and/or non-steroidal anti-inflammatory drugs (NSAIDs), proportions of patients using conventional disease modifying anti-rheumatic drugs (DMARDs; i.e., methotrexate, salazopyrine or leflunomide), and biological DMARDs. Study objectives (diagnosis or prognosis), primary outcomes, and control groups (i.e. cutaneous psoriasis, rheumatoid arthritis, spondyloarthritis, systemic lupus erythematosus, undifferentiated arthritis, osteoarthritis or healthy control (HC)) were also recorded. For all extracted data, a central value (mean or median) and variability (standard deviation or interquartile range) was collected. Study quality and risk of bias was assessed using the Newcastle Ottawa Scale for assessing the quality of non-randomized studies in meta-analysis (131).

### 2.3 Statistical analysis

Meta-analyses were performed when a diagnostic bone and cartilage turnover biomarker was reported in 2 or more different cohorts of PsA and control. Levels of biomarkers in PsA and control populations, means differences (MD) and standard deviation (SD) were extracted. If necessary, we converted median and interquartile in MD and SD using previously published methods (132). To perform sensitivity analyses, we applied a random effects models using the “one-study-removed” method as soon as there were more than two publications. Difference effect sizes were ascertained with the standardized mean difference (SMD) and its 95% confidence intervals (CI). A positive SMD confirmed a higher biomarker level in PsA than the control population. Magnitude of SMD was characterized as small (< 0.40), moderate (0.41 to 0.69) or large (> 0.70) (133).

## 3 Results

We identified 1495 records extracted from the PubMed/MEDLINE (n=514), EMBASE (n=919), and Cochrane Library (n=62) databases. After a manual search, 4 additional publications were included.

**Figure 1:**
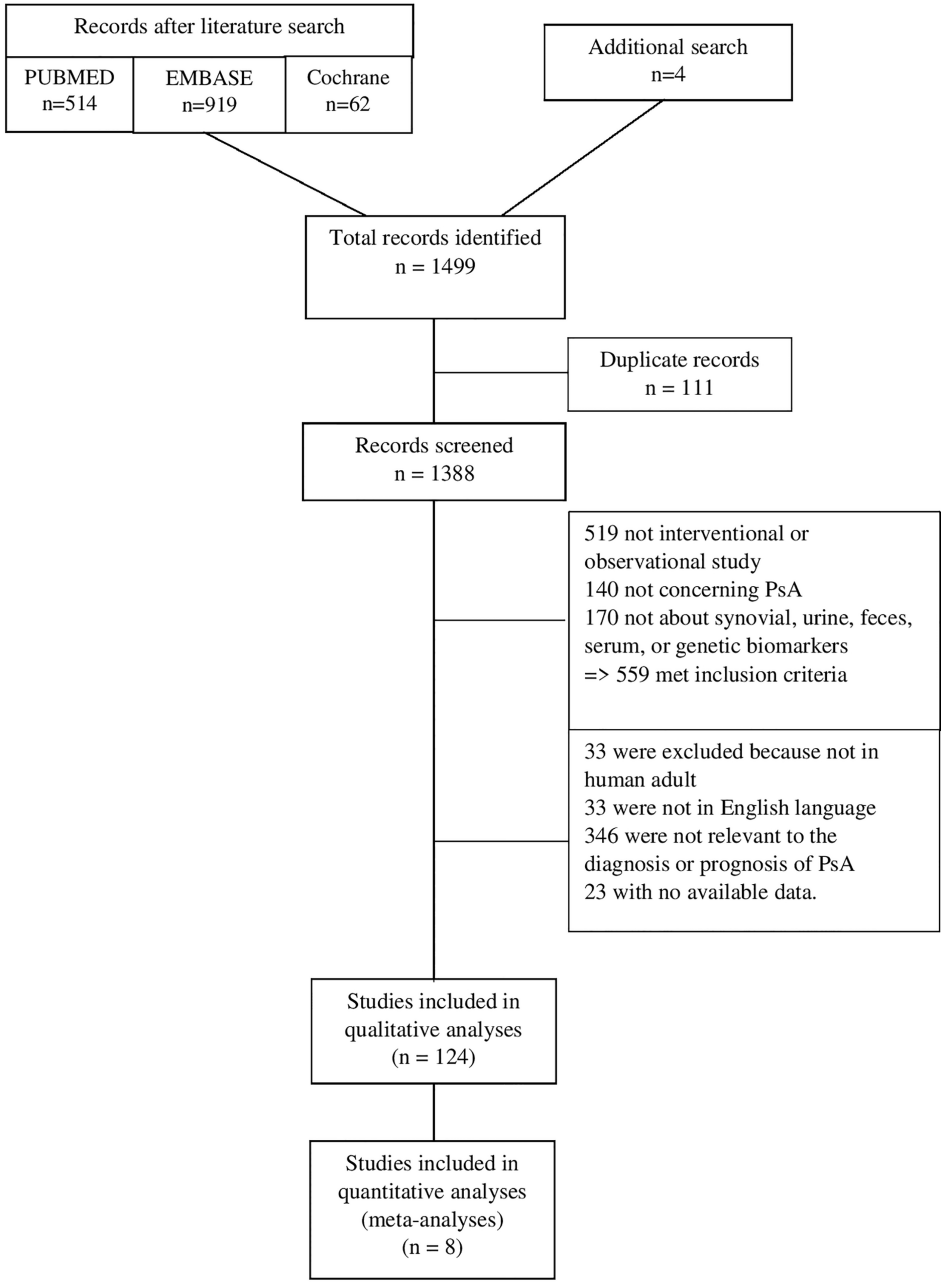
Flowchart of the study selection.

After removal of 111 duplicates, 1388 articles were screened and 559 met inclusion criteria. Subsequently, 346 publications were excluded because they did not report specific diagnostic or prognostic data for the biomarkers. Ultimately, a total of 124 studies, published from 1993 to 2022, met the eligibility criteria and were included in the qualitative analysis (**Figure 1**).

Sixty-eight studies evaluated biomarkers for diagnostic purposes, 48 evaluated biomarkers for activity or prognostic severity purposes, and 8 publications studied both diagnostic and prognostic biomarkers (**Tables 1**, **2**). An assessment of bias risk was performed for each study and is available in **Tables 3**, **4**.

**Table 1:** Included Diagnostic Biomarkers Studies.

References	Publication Year	Study Design	PsA (n)	Classification Criteria	Biomarkers	Methodology	Outcome
***Bone and cartilage turnover biomarkers***							
Mânsson (8)	2001	Cross-sectional	18	Moll & Wright	COMP; BSP; Aggrecan	Comparison of COMP, BSP and Aggrecan level by ELISA method in the synovial fluid from 18 PsA and 43 RA.	Increased levels of COMP in PsA synovial fluid compared to RA population.
Farouk (9)	2010	Cross-sectional	30	CASPAR	COMP	Comparison of COMP level using ELISA with US on 30 PsA and 30 PsO	Moderate diagnosis accuracy of COMP to distinguish PsA of PsO with an AUC of 0.56.
Chandran (10)	2010	Cross-sectional	26	CASPAR	hsCRP; IL-12; p40Il12; IL-17; RANK-L; OPG; TNFSF14; MMP3; C2C; C1-2C; CPII; COMP	Comparison between 26 PsA, 26 PsO and 26 HC of several biomarker levels using ELISA methods.	Using of biomarkers panel consisting of hsCRP, OPG, TNFSF14 and CPII:C2C ratio, PsA can be distinguished from PsO with high accuracy (AUC: 0.90).
Cretu (11)	2014	Cross-sectional	10	CASPAR	Protein expression of synovial fluid	Liquid phase chromatography with mass-spectrometry of 10 PsA and 10 OA.	CRP, MMP-3, S100A9, EPO, M2BP, DEFA1, H4, H2AFX, ORM1, CD5L, PFN1 and C4BP were overexpressed in PsA synovial tissues.
Dolcino (12)	2015	Cross-sectional	60	CASPAR	Osteopontine; osteoactivin; fibronectin1; MMP3; CathepsinZ; IL-17	Serum analysis of 60 PsA, 60 HC, 60 RA and 60 SpA after evaluation of genic expression in PBMC from 10 PsA and 10 HC.	Osteoactivin was significantly higher in PsA sera than in RA, SpA and HC sera, with 100% accuracy between PsA and HC.
Jadon (13)	2017	Cross-sectional	200	CASPAR	OPG; MMP3; Dkk-1; MCSF	ELISA determination of Dkk-1, MMP3; M-CSF and OPG levels in 200 PsA, 200 PsO, 157 SpA and 50 HC.	Biomarkers panel of Dkk-1; MCS, MMP3 and OPG was able to distinguish PsA and HC (AUC: 0.84).
Cretu (14)	2017	Cross-sectional	100	CASPAR	M2BP; CD5L; MPO; ITGB; CRP; MMP3	Level measurement by ELISA of 100 PsA, 100 PsO and 100 HC.	ITGB5, CRP and M2BP levels were increased in PsA patients (Panel of these 3 biomarkers: AUC of 0.85)
Chandran (15)	2019	Cross-sectional	73	CASPAR	COMP; hyaluronan; resistin; adiponectin; adipsin; HGF; insulin; leptin; CRP; IL-8; IL-6; IL-1β; TNFα; MCP1; NGF	Serum analysis of 77 PsA, 201 OA and 76 HC by ELISA in a first discovery phase and then comparison of 4 biomarkers in a second validation phase of 73 PsA and 75 OA.	COMP, Resistin, MCP-1 and NGF used as a panel were able to distinguish PsA and OA populations with high accuracy (AUC: 0.99).
Diani (16)	2019	Cross-sectional	50	CASPAR	MMPs; TIMPs; OPG; RANK-L; CTX; Dkk-1; SOST; CTX-I; CTX-II; PINP; Chi3L1	Serum analysis of osteoimmunologic biomarkers in 50 PsA, 50 PsO and 20 HC.	MMP2, MMP12, MMP13, TIMP2 and TIMP4 was able to distinguish PsA undergoing treatment from PsO. CHI3L1 and MMP10 was able to distinguish PsA not undergoing systemic treatment from PsO.
Waszczykowski (17)	2020	Cross-sectional	24	CASPAR	IL-18; IL-20; MMP-1; MMP-3; COMP; YKL-40; Aggrecan	ELISA analysis from 24 active PsA sera and 26 HC.	COMP, IL-18, MMP-3 and MMP-1 was able to distinguish PsA from HC.
Waszczykowski (18)	2021	Cross-sectional	22	CASPAR	IL-6; IL-18; IL-20; MMP-1; MMP-3; COMP; YKL-40; Aggrecan	ELISA analysis from 22 PsA sera, 22 OA and 23 HC.	Age-associated serum COMP and Aggrecan levels discriminate PsA from OA (AUC: 0.91)
***Genetic biomarkers***							
Elkayam (19)	2004	Cross-sectional	50	Moll & Wright	HLA	HLA class I and II typing on 50 PsA from Israeli Jewish population.	PsA was associated with HLA-A3; HLA-B13; HLA-B38; HLA-DRB0101; HLA-DRB0301; and HLA-DRB0401 on Israeli Jewish population. HLA-B27 was not associated with PsA in this cohort.
Alenius (20)	2004	Cross-sectional	120	Physician diagnosis	CTLA4; TNF; loci 8q24; 16q21 gene polymorphisms	Genotyping of TNF locus 1q21 (PSORS4) and 3q21 (PSORS5), 8q24 loci, 16q21 loci and CTLA4 locus on 120 PsA patients and 90 HC.	No association reported in this paper.
Ravindran (21)	2004	Cross-sectional	140	Moll & Wright	IL-1a; IL-1b; R-IL-1 gene polymorphisms	Genotyping of IL-1a-889; Il-1b +3953 and IL-1R+970 on 140 PsA.	Major risk allele C of IL-1a-889 loci was associated with PsA.
Batliwalla (22)	2005	Cross-sectional	19	Moll & Wright	Microarray’s analysis	Gene expression profiling on peripheral blood cells in 19 PsA patients and 19 age- and sex-matched HC.	Decreased of Nucleoporine 62KDa and MAP3K3 expression was associated with PsA compared to control.
Stoeckman (23)	2006	Cross-sectional	16	Moll & Wright	Microarray’s analysis	Whole blood gene expression profiling on 16 PsA and 15 sex- and age-matched HC.	Zinc-finger protein 395, dead box polypeptide 28, pecanex-like 3, and PI3KC2B gene expressions were upregulated in PsA compared to HC.
Butt (24)	2007	Cross-sectional	258	Physician diagnosis	VEGF; FGF1; FGF2; EGF gene polymorphisms	Genotyping with MALDI-TOF spectrophotometry on 258 PsA and 154 HC.	rs3025039*T in VEGF+936 loci was protector of PsA.
Bowes (25)	2011	Cross-sectional	1057	CASPAR	IL-13 gene polymorphism (Alleles rs20541 and rs1800925)	Genotyping of rs20541 and rs1800925 on 1057 PsA, 778 PsO and 5575 HC.	Double alleles rs1800925*C/C and rs20541*G/G were significantly associated with PsA.
Eder (26)	2011	Cross-sectional	555	Moll & Wright	IL-13 gene polymorphism (Alleles rs20541; rs843; rs1800925)	Genotyping of rs20541, rs843 and rs1800925 single nucleotide polymorphisms on 555 PsA, 342 PsO and 217 HC.	rs20541 and rs843 polymorphism increased the risk of PsA in PsO patients.
Eder (27)	2012	Cross-sectional	178 and their family	CASPAR	HLA-B and HLA-C	Family-based association study by HLA-genotyping on 178 PsA, 30 PsO and 561 first degree relatives.	HLA-B27, B-38, B-39 and HLA-C12 were associated to PsA compared to PsO.
Winchester (28)	2012	Cross-sectional	359	CASPAR	HLA-B and HLA-C	Comparison of the HLA-B and HLA-C alleles and haplotypes by HLA-genotyping on 359 PsA, 214 PsO and 1119 HC, divided in two cohort: discovery then validation.	HLA-B27 was associated to PsA.
Chandran (29)	2013	Cross-sectional	678	CASPAR	HLA alleles	HLA-genotyping on 678 PsA and 688 HC.	In comparison of HC, HLA-C*12/B*38 association, HLA-C*06/B*57 association and HLA-B*27 were associated to PsA.
Chandran (30)	2014	Cross-sectional	678	CASPAR	KIR2D and KIR3D gene polymorphism	KIR2D and KIR3D genotyping on 678 PsA and 688 HC.	The allele KIR2DS was significantly associated with PsA.
Zhang (31)	2017	Cross-sectional	465	CASPAR	36 loci, including IL-12B; RUNX3; LCE gene polymorphisms	DNA Genotyping on 465 PsA and 421 HC using MALDI-TOF spectrophotometry.	Polymorphisms in IL-12B, RUNX3 and LCE genes were associated with an increased risk of PsA.
Ciancio (32)	2017	Cross-sectional	39	CASPAR	miR-21-5p	Micro-array expression analysis of 723 miRNA on 39 PsA, 26 RA and 16 HC then PCR analysis of miR-21-5p.	miR-21-5p was overexpressed in RA and PsA population.
Cascella (33)	2017	Cross-sectional	500	CASPAR	KIF3A and IL-4 gene polymorphism	RT-PCR of rs2227282 located in the IL-4 gene and rs2285700, rs10062446 and rs2897442, located in the KIF3A gene from 500 PsA, 426 PsO and 600 HC blood samples.	Except rs2285700 on KIF3A gene, the presence of SNPs increased susceptibility to PsA but not PsO.
Abji (34)	2018	Cross-sectional	14	CASPAR	Genetic expression of Th17 pathway	RT-PCR of 84 genes from synovial fluid samples from 14 PsA, and 9 OA.	MMP3, CCL1, IL-17C, IL-3, CXCL5, IL-6 and CX3CL1 genes were expressed more in samples from PsA compared to OA.
Chen (35)	2019	Cross-sectional	111	CASPAR	HLA class I and HLA DRB1	HLA-Genotyping on 111 PsA and 207 HC from a Chinese Han population.	HLA-A*01/A*01 and HLA-C*06/C*02 were risk alleles for PsA.
Smith (36)	2020	Cross-sectional	140	CASPAR	HLA-c*06:02; B*44:02; B*27:05; B08:01; TNFRSF9; LCE3C/B; IL-23R; TNFAIP3; CSF2-P4HA2 genes	11 genes reported to be associated with PsA or PsO were genotyped in 140 PsA, 403 PsO and 181 PsO patients with joint pain.	Low accuracy of this genetic association to PsA diagnosis.
Caputo (37)	2020	Cross-sectional	424	CASPAR	SNPs of COL6A5 (rs12488457 A/C); COL8A1 (rs13081855 G/T); COL10A1 (rs3812111 A/T); miR146A (rs2910164 C/G)	Genotyping of blood sample from 424 PsA, 394 PsO and 600 HC from Italian population.	rs13081855*T, rs12488457*C and rs2910164*T were associated with PsA.
Lin SH (38)	2020	Cross-sectional	40	CASPAR	miR-941 and miR-1466-p	Expression of miR941 and miR1466-5p measured in 40 PsA, 40 PsO and 40 HC blood samples.	Higher miR-941 expression in PsA samples than PsO or HC.
Pasquali (39)	2020	Cross-sectional	28	CASPAR	Extra-vesicular micro-RNA	miRCURY™ exosome isolation kit was used to compare 14 PsA and 15 PsO blood samples in the discovery phase and then 24 PsA and 25 PsO in the validation phase.	Plasma extravesicular « let-7b-5p » and « miR-30e-5p » were significantly lower in PsA compared to PsO in validation phase.
Wade (40)	2020	Cross-sectional	31	CASPAR	Micro RNA signature	miRNA panel was assessed using miRNA Fireplex assay in sera of 31 PsA and 20 HC.	miR-221-3p, miR-130a-3p, miR-146a-3p, miR-26a-5p, miR-151a-5p and miR-21-5p were promising candidate biomarkers to distinguish PsA from HC.
Iwaszko (41)	2021	Cross-sectional	126	CASPAR	IL-33 gene polymorphisms (rs16924159; rs10975519; rs7044343)	PCR analysis of 126 PsA sera, 143 SpA, 466 RA and 229 HC.	These SNPs within the IL-33 gene were not useful for PsA diagnosis.
Cheleschi (42)	2022	Case-control	50	CASPAR	Selected mi-RNA, pro-inflammatory cytokines and adipokines	RT-PCR and ELISA analysis in blood samples from 50 PsA, 50 RA and 50 HC.	Increased expression of miR-140 and serum leptin in PsA compared to RA.
***Autoantibodies***							
Calzavara-Pinton (43)	1999	Cross-sectional	76	Moll & Wright	Anti-CCP autoantibodies	Indirect immunofluorescence test on 76 PsA sera, 38 PsO, 159 RA, 119 non- inflammatory rheumatic diseases and 204 HC.	Anti-CCP autoantibodies were specific of RA but were present in a small number of PsA cases.
Chou (44)	2010	Cross-sectional	13	CASPAR	IgG anti-agalctosyl autoantibodies	ELISA analysis on 13 PsA, 30 SpA, 22 RA and 25 HC.	IgG anti-agalactosyl autoantibodies were present in higher quantities in patients with RA, SpA and PsA serum compared to HC.
Dalmády (45)	2013	Cross-sectional	46	CASPAR	Anti-MCV autoantibodies	Serum analysis by ELISA on 46 PsA, 42 PsO and 40 HC.	Anti-MCV autoantibodies were more represented in PsA patients than in those with PsO and HC.
Dolcino (46)	2014	Cross-sectional	100	CASPAR	Anti-PsA peptide (TNRRGRGSPGAL) autoantibodies	Serum analysis of 100 PsA, 200 RA, 30 PsO, 30 LES, 30 Sjogren syndrome, 30 SpA, 30 scleroderma and 50 HC.	Anti-NRAP autoantibodies were highly associated with PsA compared to PsO, RA CCP+ or CCP-, HC and the others rheumatic diseases included.
Hu (47)	2018	Cross-sectional	12	Physician diagnosis	AC anti-SIRT1 autoantibodies	ELISA analysis on 12 PsA, 94 RA, 185 SpA and 87 HC.	Anti-SIRT1 autoantibodies were expressed higher in patients with SpA and PsA compared to patients with RA and HC but it seems to be more specific of patients with SpA.
Frasca (48)	2018	Cross-sectional	32	CASPAR	Anti-LL37 carbamylated (carb) autoantibodies and Anti-LL37 citrullinated (cit) autoantibodies	Serum analysis using ELISA from 32 PsA, 24 PsO and 12 HC.	Anti-LL37 cit were associated to psoriatic disease, while anti-LL37carb were more specific to PsA.
Yuan (49)	2019	Cross-sectional	22	Physician diagnosis	Anti-ADAMSTS5 and anti-LL37 autoantibodies	ELISA analysis of 22 PsA and 32 PsO blood samples.	IgG anti-LL37 and anti-ADAMTS5 autoantibodies distinguished PsA from PsO.
Vinci (50)	2020	Cross-sectional	69	CASPAR	IgA Anti-oxPTMCII autoantibodies	ELISA analysis on 69 PsA, 60 RA, 242 SpA, 35 PsO, 48 UA, 19 FM, and 178 HC.	IgG anti-oxPTMCII were associated with RA while IgA anti-oxPTMCII were associated with SpA, PsA and SpA associated with inflammatory bowel disease.
***Other biomarkers***							
Veale (51)	1993	Cross-sectional	15	Benett criteria	ELAM-1; ICAM-1; VCAM-1	Immunohistochemistry analysis of synovial tissue from 15 PsA and 15 RA.	Increased ELAM-1 expression in RA synovial samples compared to PsA.
Szodoray (52)	2007	Cross-sectional	43	Moll & Wright	Panel of 23 different biomarkers: VEGF, EGF; IL-10; IL-13; IFNα; MIP1α (CCL3); MIP1β (CCL4); Eotaxin (CCL11); IL12p-40	ELISA analysis on 43 PsA and 25 HC.	Overexpression of IFNα and IL-10 in PsA. Under expression of G-CSF, CCL4, CCL11, IL-13, EGF, VEGF and FGF in PsA.
Firuzi (53)	2008	Cross-sectional	16	Moll & Wright	Carbonyl (CO) and Sulfhydryl (SH) groups	Serum and Synovial analysis using spectrophotometry on 16 PsA, 18 RA and 15 OA.	Decreased SH-group in RA and PsA synovial samples compared to OA.
Hansson (54)	2014	Prospective	65	CASPAR	Calprotectin S100A8/S100A9	Serum analysis of 65 PsA and 31 HC.	ROC analysis of calprotectin S100A8/A9 revealed an AUC of 0.87 to distinguish PsA from HC.
Bosè (55)	2014	Cross-sectional	30	CASPAR	IL-2	Cytokine expression assessed on plasma circulating T-cells of 30 PsA, 21 PsO and 24 HC.	IL-2 expression was significantly associated with PsA.
Maejima (56)	2014	Cross-sectional	12	CASPAR	Moesin; K17; ANXA1; STIP-1.	Level assessment in 12 PsA, 31 PsO and 13 HC sera using dot blot analysis.	Significant increase in K17 and STIP-1 in PsA compared to PsO and HC.
Kim (57)	2015	Cross-sectional	25	CASPAR	Ratio PNN/Ly et PLQ/Ly	Compared between 25 PsA, 111 PsO, and 94 HC.	An increase in the PNN/Ly ratio and PLQ/Ly ratio was predictive of PsA.
Armas-González (58)	2015	Cross-sectional	15	CASPAR	B-cell protein expression profiling	Flow cytometry analysis on 15 PsA and 13 RA.	B-cells in RA synovial samples expressed more MHC class II molecules than those in PsA.
Amin (59)	2016	Prospective	20	Moll & Wright	RANK-L	Level measurement by ELISA of RANK-L from 20 PsA, 40 PsO and 20 HC.	Low accuracy of RANK-L to discriminate PsA from PsO (AUC: 0.66).
Gudmann (60)	2016	Cross-sectional	101	CASPAR	ProC2 and C-col10	ELISA analysis on 110 SpA, 101 PsA and 118 HC.	Increased serum ProC2 concentration in PsA and SpA.
Muntyanu (61)	2016	Cross-sectional	40	CASPAR	CXCL10	Serum analysis of 40 PsA, 14 OA, 11 RA and 8 gouts.	CXCL10 titers were higher in PsA synovial fluid than in gout and OA. No difference from RA.
Abji (62)	2016	Prospective	620	CASPAR	CXCL10	Monitoring the variation of CXCL10 titres in sera from 620 PsO.	Mean level of CXCL10 was higher in sera from PsA converter compared to non-converter.
Reindl (63)	2016	Cross-sectional	33	CASPAR	15 serum biomarkers issued in a discovery phase	ELISA analysis of serum from 33 PsA, 100 PsO and 25 HC.	Complement 3, Polymeric Immunoglobulin Receptor, Plasma Kallikrein and Zn-a2-glycoprotein were significantly higher in PsA sera compared to PsO and HC.
Alonso (64)	2016	Cross-sectional	200	Physician diagnosis	Urinary biomarker panel	Urine metabolome of 200 PsA, 200 RA, 200 PsO, 200 SLE, 200 Crohn’s disease, and 200 HC analysed using nuclear magnetic resonance.	Urine metabolome expression was different in PsA compared to RA.
Grossi (65)	2017	Cross-sectional	18	CASPAR	Calprotectin S100A8/S100A9	Assessment of serum calprotectin mean concentration from 18 PsA, 49 RA, 21 SpA and 73 HC.	High accuracy to Calprotectin S100A8/A9 to distinguish PsA from HC. No difference between PsA and SpA.
Ausavarungnirun (66)	2017	Cross-sectional	55	CASPAR	ESR and hsCRP	Inflammatory markers determination in serum from 55 PsA and 55 PsO.	Increased VS and hsCRP levels in PsA compared to PsO.
Maejima (67)	2017	Cross-sectional	11	CASPAR	VCP	Serum analysis using Reverse-phase protein array from 11 PsA, 23 PsO and 11HC.	VCP was significantly increased in PsA compared to PsO and HC.
Farrag (68)	2017	Cross-sectional	21	CASPAR	Il-34	ELISA analysis from 21 PsA, 24 PsO and 20 HC blood samples.	IL-34 concentration was able to distinguish PsA from PsO and HC (AUC: 0.90).
Sinkeviciute (69) (67)	2020	Prospective	111	CASPAR	PROM	ELISA analysis of 11 PsA and 55 HC.	PROM was associated with PsA but ROC analysis described low accuracy (AUC: 0.64).
Abji (70)	2020	Prospective	29	CASPAR	CXCL10	Monitoring of serum CXCL10 levels in 644 PsO patients to compare PsA converters (n=29) and matched non-converters (n=52).	Decrease in serum CXCL10 titers in PsA converters before and after conversion to PsA.
Esawy (71)	2020	Cross-sectional	76	Moll & Wright	Plasma Gelsolin	Serum analysis by ELISA of 76 PsA, 40 PsO and 40 age and sex-matched HC.	Gelsolin was able to discriminate PsA from PsO (AUC: 0.91) and HC (AUC: 0.98).
Souto-carneiro (72)	2020	Cross-sectional	73	Physician diagnosis	Serum metabolome and lipidome (7 lipids groups and 24 different metabolites)	Proton nuclear magnetic resonance analysis of 73 PsA sera and 49 seronegative RA.	Construction of a predictive model consisting of the lipid ratio and the expression of metabolites made it possible to distinguish RA from PsA (AUC: 0.85).
Cuervo (73)	2021	Cross-sectional	35	CASPAR	Mast-cells CD117 and fibroblasts (hsp47) in synovial fluid	Immunohistochemical analysis of cell types from synovial samples of 35 PsA, 39 RA and 31 UA (19 evolving to RA and 12 evolving to PsA).	Higher mast cell and fibroblastic density were associated with PsA progression.
Leijten (74)	2021	Cross-sectional	20	CASPAR	951 unique proteins	Serum proteomic analyses from 20 PsA samples, 20 PsO, 19 SpA and 20 HC.	No difference in proteomic expression between PsO and PsA but 68 expressed proteins differ compared to HC.
Kishikawa (75)	2021	Cross-sectional	42	CASPAR	Plasma metabolome	Plasma-metabolite profiles investigated in 42 blood samples from PsA, 50 PsO and 38 HC using dual approach by CE-TOFMS and LC-TOFMS.	In PsA compared to PsO: Increased levels of all saturated fatty acid and tyramine level. Decreased levels of mucic acid.
Fuentelsaz-Romero (76)	2021	Retrospective	9	CASPAR	Macrophage polarization	Analysis of GM-CSF expression and macrophage polarization in synovial tissue from 8 UA evolving to RA, 9 UA evolving to PsA, 16 persistent UA, 12 established RA and 10 persistent PsA.	CD163+ CD209+ macrophages were more abundant in synovial tissues from PsA and HC compared to RA and persistent UA.
Zhu J (77).	2021	Cross-sectional	4	CASPAR	Proteome profile of peripheral blood mononuclear cells	Blood samples analysis firstly using mass-spectrometry then using western-blot from 4 PsA, 4 PsO and 4 HC.	Higher SIRT2 expression in PBMC from PsA than PsO and HC.
Looby (78)	2021	Retrospective	30	CASPAR	Metabolomics	Monitoring of metabolite expression by mass spectrometry in 30 PsA, 20 PsO (10 converted to PsA and 10 non-converted to PsA), and 10 HC.	1,11-undecanedicarboxylic acid expression differed between PsA patients and HC.
Leijten (79)	2021	Cross-sectional	21	CASPAR	CD8+ CCR10+ T-cells	Flow cytometry of PBMC from 21 patients with PsA, 21 with PsO, 16 with SpA and 20 HC.	CD8+CCR10+ T-cells were more represented in PsA sera compared to HC.
Ek (80)	2021	Cross-sectional	1025	NA	21 inflammatory biomarkers	21 biomarkers were assessed in 18 different inflammatory disease populations from the UK biobank.	No biomarker measured was associated with PsA diagnosis.
Wang N (81)	2022	Cross-sectional	27	ACR criteria	Fecal metabolites	Evaluation of metabolic profile of fecal samples from 27 PsA patients, 29 with RA and 36 HC were analysed using liquid chromatography and completed by mass spectrometry.	5 fecal metabolites (α/β-turmerone, glycerol 1-hexadecanoate, dihydrosphingosine, pantothenic acid and glutamine) are potential PsA biomarkers.
Marzaioli (82)	2022	Cross-sectional	37	NA	Dendritic cells CD209/CD14+ and its cytokine expression	Flow cytometry in blood sample from 37 PsA, 62 RA, 6 OA and 11 HC and transcriptional analyses by qRT-PCR.	Higher concentration of CD209/CD14+ dendritic cells from patients with PsA and RA compared to HC. No difference of CD209+ transcriptional expression between PsA and RA.
Mc Ardle (83)	2022	Cross-sectional	95	CASPAR	Serum proteome	Comparison of protein expression from 95 PsA sera and 72 RA.	A panel of select proteins was able to distinguish PsA from RA (AUC: 0.79).

PsA, psoriatic arthritis; PsO, psoriasis; HC, Healthy control; SLE, Systemic lupus erythematosus; UA, Undifferentiated Arthritis; FM, fibromyalgia; ELISA, Enzyme-linked immune absorbent assay; RT-PCR, reverse transcription and polymerase chain reaction; MAP3K3, MAP kinase 3; PI3KC2B, phosphoinositide-3-kinase, class 2, beta polypeptide; COMP, Cartilage Oligomeric MetalloProteinase; MMP3, Matrix MetalloProteinase-3; RA, Rheumatoid arthritis; BSP, Bone Sialo-protein; AUC, Area under the curve; IL, Interleukin; OPG, Osteoprotegerin; TNFSF, Tumor Necrosis Factor Super Family; C2C, Col2-3/4 long mono; CPII, Pro-collagen 2 peptide; C1-2C, Col2-3/4 short; Pro-C2 (or PIIBNP), N-terminal propeptide of the pro-collagen IIB slice variant; Dkk1, Dikkopf-1; MCSF, Macrophage colony stimulating factor; M2BP, Mac-2 binding protein; CD5L, CD5 Like protein; MPO, Myeloperoxydase; CRP, C-reactive protein; TNF, Tumor necrosis factor; NGF, Nerve Grow Factor; Chi2L3, Chitinase like 3 protein; PROM, Matrix metalloproteinase-cleaved Prolargin; HLA, Human leukocyte antigen; CTLA-4, cytotoxic T-lymphocyte-associated protein 4; VEGF, Vascular endothelial growth factor; FGF, Fibroblast growth factor; EGF, Endothelial growth factor; ELAM1, Endothelial leukocyte adhesion molecule 1; ICAM-1, Intercellular adhesion molecule 1; VCAM-1, vascular cell adhesion molecule 1; K17, Keratin 17; ANXA1, Annexin A1; STIP-1, Stress-induced phosphoprotein-1; PNN, polynuclear; CXCL-10, CXC-motif Chemokine Ligand-10; ESR, Erythrocyte sedimentation rate; hsCRP, High sensitive C- reactive protein; VCP, Valosin-containing protein; oxPTMCII, Oxidized collagen type II; CE-TOFMS, Capillary electrophoresis time-of-flight mass spectrometry; LC-TOFMS, Liquid chromatography time-of-flight mass spectrometry.

### 3.1 Diagnostic biomarkers

#### 3.1.1 Bone and cartilage turnover biomarkers

Sixteen articles evaluated biomarkers associated with bone and cartilage turnover for their potential as PsA diagnostic biomarkers. All the studies assessing diagnostic biomarkers in our systematic review, including those assessing biomarkers panels, are listed in chronological order of publication in **Table 1**. For meta-analyses, we considered only clearly identified individual biomarkers, and not panels of biomarkers. The two mainly assessed biomarkers were Cartilage Oligometrix MetalloProteinase (COMP) and Matrix MetalloProteinase-3 (MMP3).

##### 3.1.1.1 Cartilage oligometrix metalloproteinase (COMP)

Increases of serum COMP levels by one unit resulted in an increased PsO odds ratio (OR) of 1.001 (95% CI=1.000-1.002, p=0.04), but not for PsA (OR=1.00; 95% CI=0.999-1.002, p=0.47) when comparing PsA to both PsO and HC (10). More recently, a cross-sectional study including patients with PsA, osteoarthritis (OA) and HC demonstrated in its primary discovery phase that COMP levels were significantly higher in sera of the PsA population than that of the OA population (OR=1.24; 95% CI=1.06-1.46, p=0.0062) (15). In the validation phase, serum COMP levels were not significantly different between PsA and OA populations (217.3 ng/mL vs 210.3 ng/mL, respectively p=0.344) (15). The diagnostic value of COMP was assessed in two cross-sectional studies. In the first study, serum COMP levels were significantly higher in patients with PsA (2645.3 ± 489.5 ng/mL) than in the HC population (835.9 ± 434.6 ng/mL) and clearly distinguished the 2 populations (Area Under the Curve [AUC]=0.96, 95% CI Not Available [NA]) (17). The second study compared the biomarker in PsA, OA, and HC and reported significantly higher levels in the sera of PsA patients than in the other two populations (18). COMP levels were also reported to be significantly higher in PsA synovial fluid compared to rheumatoid arthritis (RA), even in the presence of joint destruction (8).

**Figure 2:**
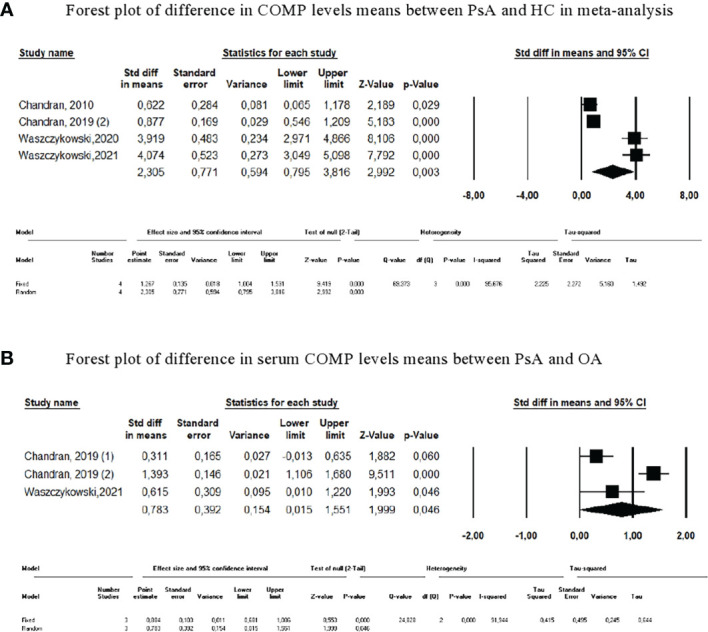
Meta-analysis of COMP levels. **(A)** Forest plot of difference in COMP levels means between PsA and HC in meta-analysis. **(B)** Forest plot of difference in serum COMP levels means between PsA and OA.

The four studies comparing serum COMP levels between PsA and HC were included in a meta-analysis (10, 15, 17, 18). COMP was significantly increased in the serum of the PsA population, and the effect size measured by SMD was 2.305 (95% CI=0.80-3.81, p=0.003; **Figure 2**). When COMP levels were compared between PsA and OA, the 3-study meta-analysis reported a significant SMD of 0.78 (95% CI=0.02-1.55, p=0.046) (15, 18).

Due to the heterogeneity of the results, sensitivity analyses were also performed. The results remained unchanged after each study was excluded serially. The difference in COMP levels between PsA and PsO was tested in only one study (10).

##### 3.1.1.2 Matrix metalloproteinase-3 (MMP3)

The results are more discordant when considering serum levels of MMP3, also known as stromelysin-1. Five cross-sectional studies revealed that MMP3 levels were significantly higher in patients with PsA than in HC (12–14, 17, 18) or in patients with PsO (13). The accuracy of MMP3 to distinguish PsA from PsO (AUC=0.70, 95% CI=0.65-0.75) and PsA from HC (AUC=0.66, 95% CI=0.59-0.74) was moderate. Other studies did not report any difference in MMP3 levels between PsA and HC (16) or PsO (14). The latter study described an increased concentration of MMP3 in PsA sera compared to PsO, with a significant OR of 1.59 (95% CI=1.21-2.11); however, this disappeared after multivariate regression (14). In a recent study, serum MMP3 levels from PsA compared to PsO and HC were not significantly different. Only one study compared PsA and OA, and did not demonstrate any difference in serum levels of MMP3 (18).

**Figure 3:**
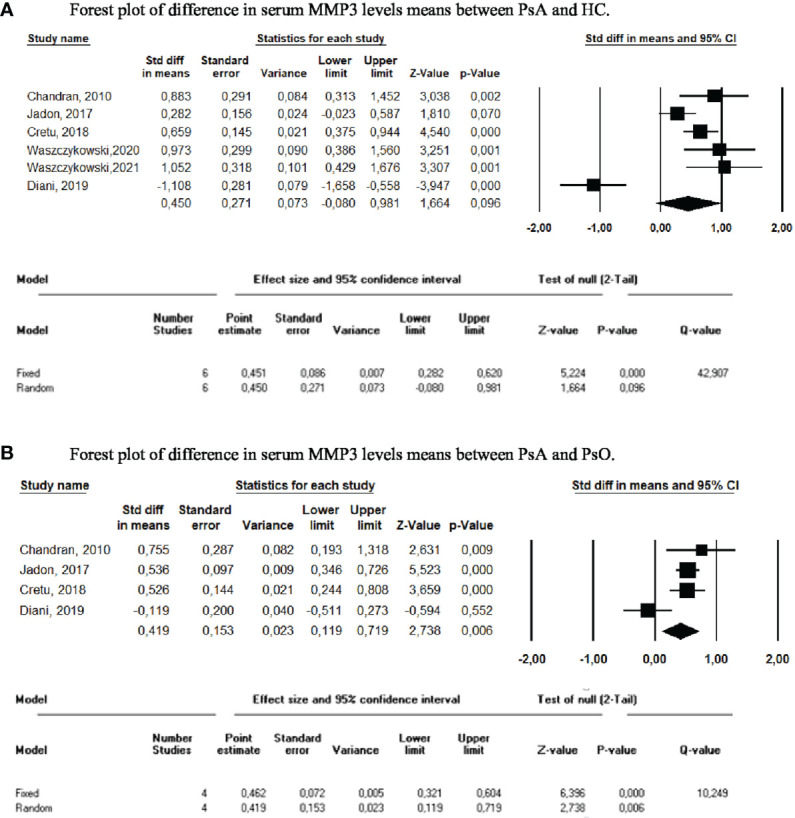
Meta-analysis of serum MMP3 levels. **(A)** Forest plot of difference in serum MMP3 levels means between PsA and HC. **(B)** Forest plot of difference in serum MMP3 levels means between PsA and PsO.

In total, 6 studies compared serum MMP3 levels between PsA and HC (10, 13, 14, 16–18) and 4 studies compared levels between PsA and PsO (10, 13, 14, 16). The pooled results displayed a higher, but not significant, level of MMP3 in sera of PsA patients compared to HC (SMD=0.450, 95% CI=-0.080-0.981, p=0.096; **Figure 3**). MMP3 was significantly higher in PsA compared to PsO (SMD=0.419, 95% CI=0.119-0.719; p=0.006). Results were confirmed with sensitivity analyses. No significant SMD was found when pooled MMP3 levels of PsO and HC were compared (SMD=-0.118 [95% CI=-0.693-0.457], p=0.689).

##### 3.1.1.3 Receptor activator of nuclear kappa-B ligand (RANK-L)

**Figure 4:**
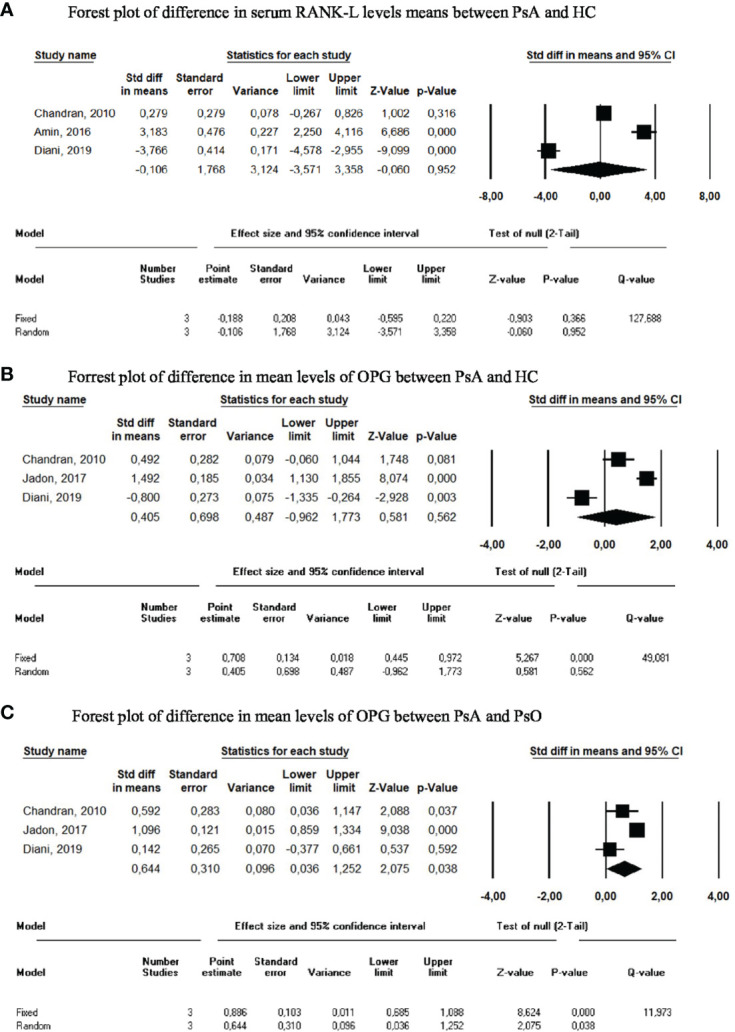
Meta-analysis of circulating biomarkers. **(A)** Forest plot of difference in serum RANK-L levels means between PsA and HC. **(B)** Forrest plot of difference in mean levels of OPG between PsA and HC. **(C)** Forrest plot of difference in mean levels of OPG between PsA and PsO.

RANK-L was also assessed as a diagnostic biomarker in three studies. Two studies reported significantly higher serum RANK-L levels in PsA patients compared with HC (59, 134), whereas the remaining study observed the opposite (16). The same three studies also compared RANK-L levels between PsA and PsO, with little or no difference detected between the two populations. The only study reporting significantly higher levels in PsA patients concluded that RANK-L was inaccurate for differentiating PsA from PsO (AUC=0.66 [95% CI NA]) (59). In the meta-analysis of these three publications, comparison of RANKL levels between PsA and HC and between PsA and PsO (10, 16, 59), the SMDs were -0.106 (95% CI=-3.75 -3.36, p=0.952) and 0.315 (95% CI=-0.391-1.021, p=0.382), respectively (**Figure 4**). Results did not change upon performance of sensitivity analyses.

##### 3.1.1.4 Osteoprotegerin (OPG)

OPG serum levels were significantly increased in a small PsA cohort (n=26 per group) compared with PsO and HC (10). However, levels were not different in a larger PsA cohort (n=200 per group) compared to PsO and HC (13), nor in another smaller PsA cohort (n=50) compared to PsO (n=50) and HC (n=20) (16). In our meta-analysis, we calculated an SMD of 0.405 (95%CI -0.962-1.773, p=0.562) when PsA was compared to HC, and an SMD of 0.644 (95%CI 0.036-1.252, p=0.038) when PsA was compared to PsO (**Figure 4**).

##### 3.1.1.5 Dickkopf-1 (Dkk-1)

**Figure 5:**
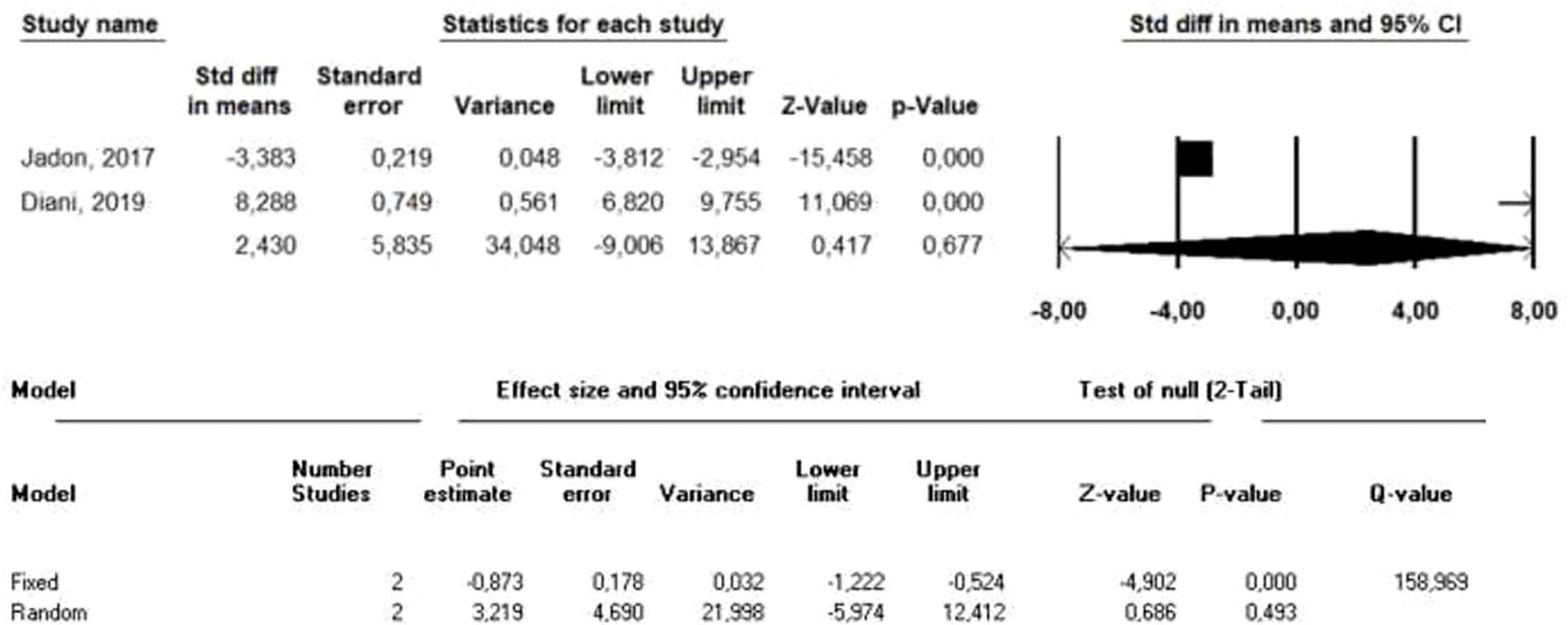
Forest plot of difference in Dkk-1 levels means between PsA and HC in meta-analysis.

Dkk-1 serum levels were studied in two publications with opposing results (13, 16). After meta-analysis, SMD in Dkk-1 levels between PsA and HC was 3.22 (95% CI=-5.974-12.412, p=0.493; **Figure 5**). When patients with PsA were compared to patients with PsO, the SMD was 0.992 (95% CI=-0.89-2.87, p=0.301).

#### 3.1.2 Genetic biomarkers

Human Leucocyte Antigen (HLA) was studied in 6 publications (19, 27–29, 35, 36). A family-based study reported an association between PsA and various HLA alleles: HLA-B*27, HLA-B*38, HLA-B*39, and HLA-C*12 (27). HLA-B*27 association with PsA was additionally described in a large cross-sectional study, while HLA-C*06 was found to be associated with skin damage and less prevalent musculoskeletal developmental phenotypes (28). Another study described an association between PsA and HLA-B*27, HLA haplotype C*12/B*38m and HLA-C*06/B*57 (29). Interestingly, HLA-B27 was not associated with PsA in the Jewish Israeli population (19), though HLA-A*01/A*01 and HLA-C*06/C*02 were risk alleles for PsA in the Chinese Han population (35).

Genetic polymorphisms were also explored in 9 different articles (20, 21, 24–26, 30, 31, 33, 41). Polymorphisms of the gene Il-13 were examined in two studies, which reported that rs1800925*C and rs20641*G were significantly associated with PsA in a PsO population (25, 26). However, one study reported the association between rs1800925 polymorphism and PsA only in the smoker population (26).

#### 3.1.3 Autoantibodies

In our systematic literature review, autoantibodies were explored in 8 articles (43–50). They focused on different autoantibodies, preventing meta-analysis. Among these autoantibodies, those of interest are outlined below. It should be noted that none of these autoantibodies have been evaluated or validated in any additional PsA cohorts beyond those described here.

Anti-Cyclic citrullinated peptides (CCP) were present in some PsA patients (7.9%) (43). Anti-Mutated citrullinated vimentin (MCV) levels were significantly higher in PsA sera (24%) than PsO (8%) (45). The novel autoantibody named anti-PsA peptide was identified after screening of a random synthetic peptide library with pooled immunoglobulins derived from 30 patients with recent onset PsA. Anti-PsA shares a sequence homology with Nebullin Related Anchoring Protein (N-RAP) (46). A peptide corresponding to N-RAP sequence was synthetized and tested by ELISA. Anti-NRAP autoantibodies were recognized by 83% of PsA sera, versus 7% of rheumatoid arthritis (RA) anti-CCP positive, 4% of RA anti-CCP negative, 3.3% of PsO, and none of other rheumatic diseases included in this study (46).

Anti-A Disintegrin and MetalloproteinaSe with ThromboSpondin motifs 5 (ADAMSTS5) and anti-Cathelicidin LL37 (LL37) IgG autoantibodies were assessed for differentiating PsA from PsO. The ROC analysis reported an AUC of 0.84 (95% CI=NA, p<0.1) for anti-ADAMSTS5 autoantibodies and 0.87 (95% CI=NA, p<0.01) for anti-LL37 autoantibodies (49). Expression of Carbamethylated anti-LL37 was significantly higher in PsA sera (median=0.66, IQR=0.439) than PsO (median=0.43, IQR=0.47, p=0.02) and HC (median=0.158, IQR=0.099, p=0.0001) (48). Finally, higher IgA anti-oxidized collagen type II (oxPTMCII) autoantibodies were detected in PsA (84%, n=33/39) and axial spondyloarthritis (SpA, 47%, n=79/165) sera compared to HC (0%, n=0/28) (50).

#### 3.1.4 Other biomarkers

Two additional biomarkers unrelated to the above categories were also assessed in at least 2 publications.

##### 3.1.4.1 C-X-C motif chemokine ligand 10 (CXCL10)

CXCL10 rates were overexpressed in synovial fluid of PsA versus gout or SpA, but rates were similar to those of RA (61). A prospective follow-up of patients with PsO reported both higher baseline serum CXCL10 levels in patients who subsequently developed PsA as compared with those who did not, and a significant decrease in CXCL10 levels from the year before to the year after PsA onset (70).

##### 3.1.4.2 Calprotectin S100A8/S100A9

A serum calprotectin S100A8/S100A9 with a cut-off of 475 ng/mL was able to discriminate PsA from HC with a 93.3% specificity and 75.0% sensitivity (54). Serum calprotectin levels were increased in PsA but also in other inflammatory arthritis (RA, axial SpA) compared to controls (i.e., OA, fibromyalgia, and undifferentiated arthralgia), with good accuracy for distinguishing inflammatory arthritis from controls (AUC=0.964, 95% CI=NA) (65). Calprotectin levels in each subpopulation were not available for a meta-analysis.

### 3.2 Prognostic biomarkers

Prognostic biomarkers, including markers of disease activity, were less frequently assessed than diagnostic biomarkers (**Table 2**). As such, the data collected were insufficient to perform meta-analyses.

**Table 2:** Included Prognostic Biomarkers Studies.

References	Publication year	Study design	PsA (n)	Classification criteria	Biomarkers	Methodology	Outcome
***Acute Phase Reactant Biomarkers***							
Helliwell (84)	1991	Cross-sectional	36	Moll & Wright	Cytidine deaminase; ESR; CRP; histidine	Concentration assessment in 36 PsA blood samples.	Better correlation with disease activity was ESR.
Mchugh (85)	2003	Prospective	87	Moll & Wright	ESR	5-year follow-up of a cohort of 87 PsA patients.	Correlation between high initial ESR level and pejorative disease progression.
Bandinelli (86)	2015	Cross-sectional	112	CASPAR	ESR; CRP; Anti-CCP; HLA	US examination, HLA typing and serum analysis of 112 patients with PsA.	US abnormalities were associated with higher mean serum CRP or ESR levels and expression of HLA-B27, B-35, B38, Cw*6 and DR4.
***Bone and cartilage biomarkers***							
Kane (87)	2003	Retrospective	22	Moll & Wright	MRP8; MRP 14	ELISA analysis of synovial and serum sample from 22 patients with PsA, 11 with RA 15 with SpA.	Serum MRP8 and MRP14 concentrations were markers of disease activity in PsA, RA and SpA.
Skoumal (88)	2008	Cross-sectional	64	Moll & Wright	COMP	ELISA analysis of serum from 64 patients with PsA and 39 with PsO.	COMP concentration was significantly correlated with CRP, ESR, TCJ and SCJ.
Dalbeth (89)	2010	Cross-sectional	38	CASPAR	Dkk-1; M-CSF; RANK-L; OPG	Serum analysis from 38 PsA, 10 PsO and 12 HC.	RANK-L and M-CSF are correlated with articular radiographic damage in PsA.
Van kuijk (90)	2010	Prospective	24	CASPAR	CPII; PINP; MIA; MMP-3; C2C; COMP; osteocalcin; NTX-1; CTX-1	Serum analysis using ELISA for 12 weeks follow-up of 24 PsA in an adalimumab study.	Decreased MMP-3 concentration was associated with DAS28 improvement.
Ramonda (91)	2013	Prospective	43	CASPAR	MMP3; VEGF; PTX3; hsCRP	ELISA analysis on 43 HC and 43 PsA at inclusion and 24 weeks after anti-TNF introduction.	Decrease in biomarker levels during follow-up.
Waszczykowski (18)	2021	Cross-sectional	24	CASPAR	IL-18; IL-20; MMP-1; MMP-3; COMP; YKL-40; Aggrecan	ELISA analysis from 24 patients with active PsA sera and 26 HC.	COMP level was significantly correlated with TJC and SJC.
Chung (92)	2021	Cross-sectional	69	CASPAR	Dkk-1	ELISA analysis from 69 PsA sera, 39 RA and 21 HC. Radiographic hands erosions and sacroiliitis were assessed.	Bone erosions, sacroiliitis and SCJ were correlated with elevated serum levels of Dkk-1.
***Genetic biomarkers***							
Alenius (93)	2002	Cross-sectional	88	Moll & Wright	HLA-A; HLA-B; HLA-Cw*; HLA-DRB1*; HLA-DQB1*	HLA Genotyping of 88 PsA and 1085 HC.	HLA-B37 and HLA-B62 were markers of erosions and deformations in PsA.
Iwaszko (41)	2021	Cross-sectional	126	CASPAR	IL-33 gene polymorphisms (rs16924159; rs10975519; rs7044343)	PCR analysis in 126 PsA, 143 AS, 466 RA and 229 HC.	No correlation between SNPs within IL-33 gene and CRP or BASDAI in PsA.
***Autoantibodies***							
Korendowych (94)	2005	Cross-sectional	126	Physician diagnosis	Anti-CCP	Immunofluorescence analysis from 126 PsA sera.	All PsA CCP+ had radiographic damages and significantly higher SJC.
Perez-alamino (95)	2014	Prospective	81	CASPAR	Anti-CCP	ELISA assessment from 81 PsA sera.	PsA CCP+ had more erosive damage and higher polyarticular forms.
Ibrahim (96)	2018	Cross-sectional	45	CASPAR	Anti-CarP autoantibodies	ELISA analysis of sera from 45 patients with PsA and 45 HC.	Anti-CarP autoantibodies were associated to both clinical and US activity in PsA.
Frasca (48)	2018	Cross-sectional	32	CASPAR	Anti-LL37 carbamylated autoantibodies and Anti-LL37 citrullinated autoantibodies	ELISA analysis from 32 PsA sera, 24 PsO, and 12 HC.	Correlation with DAS44 in PsA.
***Other biomarkers***							
Elkayam (97)	2000	Cross-sectional	34	Moll & Wright	Hyaluronic acid	Radiometric assay of sera 34 patients with PsA, 34 with RA and 49 with HC.	Hyaluronic acid level was correlated to PASI Score (r: 0.87).
Elkayam (98)	2000	Cross-sectional	34	Moll & Wright	IL-10; IL-6; IL-1ra; IL-2R	ELISA analysis of serum samples from 34 patients with PsA and 10 HC.	Increase in IL-1Ra titers associated with joint activity in PsA.
Foell (87)	2003	Prospective	22	Moll & Wright	Calprotectin S100A12	Serum analysis before and after MTX introduction of 22 PsA patients, 9 RA patients, 11 SpA patients and 30 HC.	Calprotectin S100A12 concentration was modestly correlated with ESR and Richie index.
Rooney (99)	2004	Prospective	5	Moll & Wright	IL-18	Monitoring expression in synovial tissues from 5 patients with PsA, 11 with RA, 9 with UA and 2 with reactive arthritis before MTX or Salazopyrine introduction, repeated after 12 months delay.	IL-18 expression correlated with CRP circulating levels in all groups before treatment. Decrease expression of IL-18 after treatment correlated with a decrease in CRP levels.
Fink (100)	2007	Cross-sectional	28	Moll & Wright	VEGF	ELISA analysis of sera from 28 patients with PsA and 9 HC.	Higher VEGF titres in the active PsA subgroup.
Madland (101)	2007	Cross-sectional	119	Physician diagnosis	ESR; hsCRP; Calprotectin S100A8/A9; Calprotectin S100A12	Serum analysis from 119 PsA patient blood samples.	Calprotectin S100A8/A9 was inferior to ESR and CRP to assess disease activity but was a better marker of radiographic damage.
Szodoray (52)	2007	Cross-sectional	43	Moll & Wright	Panel of 23 different biomarkers	ELISA analysis from 43 patients with PsA sera and 25 HC.	EGF, IFNα, VEGF, CCL3 and IL-12 p-40 levels correlated with disease activity.
Alenius (102)	2009	Cross-sectional	134	Moll & Wright	IL-6; IL-2R	Serum analysis from 134 patients with PsA and 85 PsO patients using ELISA.	IL-6 is associated to ESR, CRP and TCJ.
Pongratz (103)	2010	Cross-sectional	52	CASPAR	BAFF and testosterone	Serum analysis from 53 PsA patient blood samples.	In male patients with PsA, the BAFF/testosterone ratio was significantly correlated with DAS28.
Celis (104)	2011	Cross-sectional	46	CASPAR	miRNA expression and panel of 21 cytokines.	ELISA analysis associated to quantitative RT-PCR of synovial tissues from 46 PsA.	Association between synovial IL-6 and CCL20 levels and circulating CRP levels.
Przepiera-będzak (105)	2013	Cross-sectional	80	CASPAR	VEGF; EGF; FGFβ; FGFα	Serum analysis on 80 PsA patient samples, 18 with SAPHO and 20 HC.	VEGF and CRP are correlated. VEGF is not associated with several disease activity score.
Jensen (106)	2011	Prospective	42	Moll & Wright	YKL-40 (Chi3l1); hsCRP	Serum analysis on 42 patients with PsA and 6 with PsO before and after adalimumab introduction.	YKL40 is decreased in responder PsA patients.
Eissa (107)	2013	Cross-sectional	50	CASPAR	Kallikreins	Serum analysis on 50 patients with PsA, 50 PsO patients and 26 HC.	Kallikrein 8 and Kallikrein 6 are associated with PASI in PsA patients.
Kayikci (108)	2013	Cross-sectional	48	CASPAR	Il-17; Il-22; Il-23	ELISA analysis on 48 patients with PsA, 20 with PsO and 19 HC.	Il-17 is weakly correlated with TJC.
Hansson (54)	2014	Prospective	65	CASPAR	Calprotectin S100A8/S100A9	ELISA analysis in sera from 65 patents with PsA and 31 HC.	Calprotectin was significantly increased in polyarticular form of PsA.
Ahmed (109)	2015	Cross-sectional	26	CASPAR	YKL-40 (Chi3l1)	ELISA analysis of blood samples from 26 pateints with PsA, 22 with PsO and 30 HC.	YKL40 was significantly correlated with CPDAI in PsA patients.
Husakova (110)	2015	Cross-sectional	40	Physician diagnosis	Prolactin	Immunoradiometric assay on sera of 70 patients with PsO, 40 with PsA and 27 HC.	No correlation was found with disease activity.
Matt (111)	2015	Cross-sectional	23	CASPAR	Fc receptor of Monocyte-cells	Flow cytometru on sera of 23 patients with PsA and 32 HC.	High titer of CD64+ cells was significantly correlated with DAS28.
Peled (112)	2015	Cross-sectional	20	CASPAR	PD1	Flow cytometry on 20 patients with PsA and 15 HC.	A low titre of T-cell presenting PD1 is associated with a low DAS28 score.
Dikbas (113)	2016	Cross-sectional	28	CASPAR	Adipocytokine: Visfatin; Resistin; Adiponectin	ELISA analysis on 28 PsA patients and 39 HC.	No correlation found.
Munk (114)	2016	Cross-sectional	101	CASPAR	PIIANP; C2M	ELISA analysis of 110 patients with SpA, 101 with PsA and 96 HC.	No correlation found.
Alonso (64)	2016	Cross-sectional	200	Physician diagnosis	Urinary biomarker panel	Nuclear magnetic resonance analysis of urine samples from 200 patients with PsA, 200 with RA, 200 with PsO, 200 with SLE, 200 with Crohn’s disease, and 200 HC.	Increased urine levels of citrate in active PsA.
Inciarte-mundo (115)	2016	Cross-sectional	50	CASPAR	Calprotectin and TNF trough serum levels	Serum analysis from 50 patients with PsA and 42 with RA.	High Calprotectin titers were associated with US activity in both PsA and RA patients.
Przepiera (116)	2016	Cross-sectional	76	CASPAR	IL-18; Fetuin A; ICAM-1; ET-1	ELISA analysis of sera from 76 patients with PsA sera, 81 with SpA and 34 with SAPHO.	ET-1 levels were correlated to DAS28 in PsA. IL-18 levels were associated to BASDAI.
Kiliç (117)	2017	Cross-sectional	116	CASPAR	MPV	Serum analysis from 116 PsA patients, 41 PsO patients and 90 HC.	Skin disease severity was associated to high MPV concentration in patients with PsA.
Farrag (68)	2017	Cross-sectional	21	CASPAR	IL-34	ELISA assessment in sera from 21 patients with PsA, 24 with PsO and 20 HC.	Association between IL-34 and disease articular and PASI, CPDAI, and BASDAI.
Inciarte-mundo (118)	2018	Prospective	51	CASPAR	Calprotectin; TNF trough serum levels	Serum analysis at baseline and relapse of 47 PsA patients and 56 with RA treated with anti-TNF.	High Calprotectin concentration at baseline was associated with relapse in both PsA and RA patients.
Wade (119)	2018	Cross-sectional	N.A.	N.A.	Polyfunctional T-cells	Flow cytometry analysis on patients with PsA.	Th1 and Th17 pathways were associated with DAPSA.
Coras (120)	2019	Cross-sectional	41	CASPAR	Eicosanoids	Assessment using liquid phase chromatography and mass spectrometry on 41 patients with PsA.	9 pro-inflammatory eicosanoids were associated to DAS28 while 18 anti-inflammatory eicosanoids were inversely correlated with it.
Colak (121)	2019	Cross-sectional	50	CASPAR	VASPIN; NGAL; Apolipoprotein	Serum analysis on 50 patients with PsA and 36 HC.	No significant correlation found with disease activity.
Sakellariou (122)	2019	Cross-sectional	78	CASPAR	Calprotectin	ELISA analysis from 78 patients with PsA sera and 78 with RA.	Modest correlation with US activity score.
Coras (123)	2019	Cross-sectional	38	CASPAR	Cholin metabolites	Liquid phase chromatography and mass spectrometry analysis on 38 PsA.	Trimethylamine-N-oxide was correlated to DAS28, CPDAI and BSA.
Ozisler (124)	2020	Retrospective	47	CASPAR	RDW	Blood samples analysed from 47 patients with PsA and 56 HC.	RDW was modestly correlated to DAS28, CRP and ESR in PsA patients.
Arias de la rosa (125)	2020	Cross-sectional	80	CASPAR	Complement C3	Serum analysis on 80 patients with PsA, 200 with RA, 150 with SpA and 100 HC.	Higher C3 concentration in PsA population with DAS28> 5.1.
Esawy (71)	2020	Cross-sectional	76	CASPAR	Plasma Gelsolin	ELISA analysis of sera from 76 patients with PsA, 40 with PsO and 40 age- and sex-matched HC.	Negative correlation between Gelsolin titers and DAPSA or CPDAI.
Jarlborg (126)	2020	Cross-sectional	237	CASPAR	Calprotectin S100A8/S100A9	Serum analysis on 969 RA patient blood samples, 451 patients with SpA, 237 PsA patients and 72 HC.	No correlation founded with disease activity in PsA patients.
Medvedeva (127)	2020	Prospective	80	N.A.	155 different proteins	Immunoassay on sera from 80 pateints with PsA, 175 with SpA and 93 with PsO during different apremilast’s phase 3 studies.	No correlation founded with disease activity in PsA patients.
Wcisło-dziadecka (128)	2020	Prospective	6	CASPAR	TNF- α; TNFR1; TNFR2	Mass spectrometry analysis from blood samples in 6 patients with PsA treated with adalimumab.	Serum TNF higher titers was associated with lower DAS28.
Boyd (129)	2020	Cross-sectional	45	CASPAR	Panel of 12 biomarkers	Immunoassays analysis in blood samples from 45 patients with PsA.	Moderate correlation between YKL-40, IL-6, ICAM-1, and composite score (SAA CDAI, SDAI, DAS28, HAQ and MDA).
Leijten (74)	2021	Prospective	20	CASPAR	951 unique proteins	Serum proteomic analyses from 20 patients with PsA, 20 with PsO, 19 with AS and 20 HC.	ICAM-1 and CCL-18 levels were correlated with SCJ. PI3 correlated with PASI score.
Coras (130)	2021	Cross-sectional	19	CASPAR	Oxylipins profile	Reverse-phase chromatography analyses in sera from 19 patients with PsA and 20 with PsO.	Oxylipins profile correlated with skin activity in PsO and PsA patients but not with articular activity in PsA patients.

PsA, psoriatic arthritis; PsO, psoriasis; HC, Healthy control; OA, Osteoarthritis; RA, Rheumatoid arthritis; LES, lupus erythematosus systemic; UA, Undifferentiated Arthritis; SpA, Spondyloarthritis; SAPHO, Synovitis Acne Pustulosis Hyperostosis Osteitis; CASPAR, Classification criteria for Psoriatic Arthritis; PASI, Psoriatic Area and Severity Index; BSA, Body Surface Area; DAS, Disease Activity Score; CPDAI, composite psoriatic disease activity index; DAPSA, Disease Activity in PSoriatic Arthritis; BASDAI, Bath Ankylosing Spondylitis Disease Activity Index; ELISA, Enzyme-linked immune absorbent assay; MTX, Methotrexate; MRP, Myeloid Related Protein; IL, Interleukin; TNFSF, Tumor Necrosis Factor Super Family; CRP, C-reactive protein; TNF, Tumor necrosis factor; HLA, Human leukocyte antigen; VEGF, Vascular endothelial growth factor; Anti-CCP, Anti-cyclic citrullinated protein; anti-LL37, anti-cathelecidin; Anti-ADAMTS5, A disintegrin and metalloproteinase with thrombospondin motifs; ICAM-1, Intercellular adhesion molecule 1; ESR, Erythrocyte sedimentation rate; hsCRP, High sensitive c reactive protein; IFN, Interferon; COMP, Cartilage Oligomeric MetalloProteinase; TCJ, Tender Joint Count; SCJ, Swollen Joint Count; Blys, B lymphocyte Stimulator; BAFF, B cells activating factor belonging to TNF Family; M-CSF, Macrophage colony stimulating factor; MIA, melanoma inhibitory activity; NTX, N-terminal cross-linked telopeptide of type I collagen; ITCP, C-telopeptide of Type I Collagen; RT-PCR, reverse transcription and polymerase chain reaction; CCL-x, chemokine ligand-x; Chi3L1, Chitinase 3 like protein; PTX3, Pentraxin 3; PIIANP, procollagen IIA N-Terminal peptide; C2M, Matrix metalloproteinase-generated type II collagen fragment; MPV, Mean platelet volume; PD1, Programmed Death-1; ET-1, Endothelin 1; VASPIN, visceral adipose tissue-derived serine protease inhibitor; NGAL, Neutrophil-gelatinase associated lipocalin; Anti-CarP, Anti-Carbamethylated protein; RDW, Red blood cell distribution width; MMP3, Matrix MetalloProteinase-3; MDA, Minimal activity disease.

#### 3.2.1 Acute phase reactant biomarkers

One study concluded that erythrocyte sedimentation rate (ESR) was better correlated with Ritchie’s index, tender joint count (TJC) and swollen joint count (SJC) than C-Reactive Protein (CRP) in a 36 patient PsA cohort (84). These potential prognosis markers were explored in a 5-year follow-up prospective study. The 36 patients with PsA demonstrated a disease duration ranging from 1-40 years, and baseline ESR was associated with structural progression (85). Both ESR and CRP were also associated with UltraSonography (US) which are signs of active synovitis (86).

#### 3.2.2 Bone and cartilage turnover biomarkers

Serum COMP levels were correlated with acute phase reactants and disease activity (TJC, r=0.60, p<0.001) and SJC, r=0.75, p<0.0001) (18, 88). A decrease in MMP3 serum levels in PsA patients receiving adalimumab was correlated with Disease Activity Score (DAS)-28 improvement (90).

#### 3.2.3 Autoantibodies

In a cross-sectional study, positivity for anti-CCP antibodies was associated with more radiographic damage and polyarticular phenotypes (95). Anti-LL37 autoantibodies were also described to correlate with disease activity in PsA (48). A strong correlation between Anti-Carbamethylated Protein (CarP) antibody levels and both clinical and ultrasonographic activity was described (correlation between anti-CarP and DAS-28 (r=0.96), CRP (r=0.97), ESR (r=0.97) and US power Doppler+synovitis with a Pearson coefficient >0.97) (96).

#### 3.2.4 Other biomarkers

##### 3.2.4.1 Serum calprotectin S100A8/A9

Calprotectin S100A8/A9 plasma levels were not correlated with disease activity (122, 126). Although, one study reported calprotectin S100A8/A9 levels significantly increased in the polyarticular phenotype of PsA, and were correlated with SJC (54). Contradictory results were reported regarding correlation with US synovitis in greyscale and power Doppler analyses (115, 122). High levels of calprotectin were also associated with relapse at 1-year (118).

##### 3.2.4.2 YKL40

Serum concentration of YKL40, also named Chitinase like-3 protein (Chi3L1) was significantly correlated with disease activity (r=0.848, p<0.001) (109). It was also sensitive to changes, with a significant decrease in serum of good responders to TNF inhibitor (106).

**Table 3:** Assessment of bias risk using NOS scale for case-control studies.

	Selection				Comparability	Exposure		
References	Is the case definition adequate?	Representatives of the case	Controls selection	Controls definition	Comparability of cases and controls based on the design or analysis	Ascertainment of exposure	Same method of ascertainment for cases and controls	Non-response rate
Abji, 2017 (34)	A*	A*	B	A*	A*	D	A*	C
Ahmed, 2014 (109)	A*	B	A*	A*	AB**	D	A*	C
Alenius, 2002 (93)	A*	B	B	A*		D	A*	C
Alenius, 2004 (20)	A*	B	A*	B	B*	D	A*	C
Alenius, 2009 (102)	A*	B	B	A*		D	A*	C
Alonso, 2016 (64)	C	B	B	B	B*	D	A*	C
Amin, 2016 (59)	A*	A*	C	A*	A*	D	A*	C
Arias de la rosa, 2020 (125)	A*	B	B	A*	A*	D	A*	C
Armas-Gonzales, 2015 (58)	A*	B	B	A*		D	A*	C
Ausavarungnirun, 2017 (66)	A*	A*	B	A*	AB**	D	A*	C
Bandinelli, 2015 (86)	A*	B		B		D	A*	C
Batliwalla, 2006 (22)	A*	B	A*	B	A*	D	A*	C
Bosè, 2014 (55)	A*	B	B	B		D	A*	C
Bowes, 2011 (25)	A*	A*		B	B*	D	A*	C
Boyd, 2020 (129)	A*	A*	A*	B		D	A*	C
Butt, 2007 (24)	A*	A*	A*	A*	B*	D	A*	C
Calzavara, 1999 (43)	A*	B	C	B		D	A*	C
Caputo, 2020 (37)	A*	A*	B	A*	A*	D	A*	C
Cascella, 2017 (33)	A*	A*	B	A*		D	A*	C
Celis, 2011 (104)	A*	B				D		C
Chandran, 2010 (10)	A*	A*	B	A*	AB**	D	A*	C
Chandran, 2014 (30)	A*	B	B	A*	A	D	A*	C
Chandran, 2019 (15)	A*	B	B	A*	A*	D	A*	C
CheleschI, 2021 (42)	A*	A*	B	A*	A*	C	A*	C
Chen, 2019 (35)	A*	B	C	A*	B*	D	A*	C
Chou, 2010 (44)	A*	A*	B	A*	A	D	A*	C
Chung, 2021 (92)	A*	A*	C	A*	A*	D	A*	C
Ciancio, 2017 (32)	A*	B	C	A*		D	A*	C
Colak, 2018 (121)	A*	A*				D		
Coras, 2019 (120)	A*	B				D		
Coras, 2019 (123)	A*	B				D		
Coras, 2021 (130)	A*	A*	A*	B	A*	D	A*	C
Cretu, 2014 (11)	A*	B	B	A*	AB**	D	A*	C
Cretu, 2018 (24)	A*	B	A*	A*	B*	D	A*	C
Cuervo, 2021 (73)	A*	A*	B	A*	B*	D	A*	C
Dalbeth, 2010 (89)	A*	B	B	A*		D	A*	C
Dalmády 2013 (45)	A*	A*	A*	A*		D	A*	C
Diani, 2019 (16)	A*	A*	C	B	A*	D	A*	C
Dikbas, 2014 (113)	A*	B	C	B		D	A*	C
Dolcino, 2014 (46)	A*	B	C	A*	B*	D	A*	C
Dolcino, 2015 (12)	A*	A*	C	A*	AB**	D	A*	C
Eder, 2011 (26)	A*	A*	A*	A*		D	A*	C
Eder, 2012 (27)	A*	B	B	B		D	A*	C
Eissa, 2013 (107)	B	B	B	B	AB**	D	A*	C
Ek, 2021 (80)	B	A*	B	A*	A*	D	A*	C
Elkayam, 2000 (98)	A*	B		B		D	A*	C
Elkayam, 2000 (97)	A*	B	C	B		D	A*	C
Elkayam, 2004 (19)	A*	A*	C	B		D	A*	C
Esawy, 2020 (71)	A*	A*	B	A*		D	A*	C
Farouk, 2010 (9)	A*	A*	A*	B	AB**	D	A*	C
Farrag, 2017 (68)	A*	A*	C	A*	AB**	D	A*	C
Fink, 2007 (100)	A*	B	C	A*		D	A*	C
Firuzi, 2008 (53)	A*	B	C	A*		D	A*	C
Foell, 2003 (87)	B	B	C	B		D	A*	C
Frasca, 2018 (48)	A*	B	B	A*	A*	D	A*	C
Grossi, 2017 (65)	A*	A*	C	A*		D	A*	C
Gudmann, 2016 (60)	A*	A*	A*	B	B*	D	A*	C
Hansson, 2014 (54)	A*	B	C	B	AB**	D	A*	C
Helliwell, 1991 (84)	A*	B				D		
Hu, 2018 (47)	A*	B	C	A*	A*	D	A*	C
Husakova, 2015 (101)	A*	B	C	B	A*	D	A*	C
Ibrahim, 2017 (96)	A*	B	C	B	A*	D	A*	C
Inciarte-Mundo, 2016 (115)	A*	B	B	A	B*	D	A*	C
Jadon, 2017 (13)	A*	A*	A*	A*	AB**	D	A*	C
Jarlborg, 2020 (126)	B	A*	B	A*	A*	D	A*	C
Jensen, 2012v (106)	A*	B	B	A*	A*	D	A*	C
Kane, 2003 (87)	A*	B	B	A*		D	A*	C
Kayikci, 2013 (97)	A*	A*	C	B		D	A*	C
Kim, 2015 (57)	A*	A*	B	A*	AB**	D	A*	C
Kishikawa, 2021 (75)	A*	A*	B	B	A*	C	A*	C
Korendowych, 2005 (94)	A*	B				D		
Leijten E, 2021 (74)	A*	A*	B	B	A*	C	A*	C
Leijten EF, 2021 (79)	A*	A*	B	A*	A*	C	A*	C
Lin, 2020 (38)	A*	B	C	A*		D	A*	C
Looby, 2021 (78)	A*	A*	B	A*	A*	D	A*	C
Madland, 2007 (101)	A*	B				D		
Maejima, 2014 (56)	A*	B	C	A*		A*	A*	C
Maejima, 2017 (67)	B	A*	C	B		D	A*	C
Maejima, 2017 (118)	A*	B	B	B		D	A*	C
Mansson, 2001 (8)	A*	A*	A*	A*		D	A*	C
Marzaioli, 2022 (82)	A*	A*	B	A*	A*	D	A*	C
Matt, 2015 (111)	A*	B	C	B	A*	D	A*	C
Mc Ardle, 2021 (83)	A*	A*	B	A*	A*	D	A*	C
McHugh, 2003 (85)	A*	B				A*		
Medvedeva, 2020 (127)	A*	B	B	A		A*	A*	C
Munk, 2015 (114)	A*	B	C	B	A*	D	A*	C
Muntyanu, 2016 (61)	A*	A*	B	A*		D	A*	C
Ozisler, 2019 (124)	A*	B	C	B	A*	D	A*	C
Pasquali, 2020 (39)	A*	A*	B	A*	A*	D	A*	C
Peled, 2015 (112)	A*	B	B	A*	B*	D	A*	C
Perez-Alamino, 2014 (95)	A*	B				D		
Pongratz, 2010 (103)	A*	A*				D		
Przepiera-Będzak, 2013 (105)	A*	B	C	B	A*	D	A*	C
Przepiera-Będzak, 2016 (116)	A*	B	C	B	A*	D	A*	C
Ramonda, 2013 (91)	A*	A*	C	B	A*	D	A*	C
Ravindran, 2004 (21)	A*	A*	A*	B		D	A*	C
Reindl, 2016 (63)	A*	B	C	B		D	A*	C
Rooney, 2004 (99)	A*	B	C	B		D	A*	C
Sakellariou, 2019 (122)	A*	B	B	B	A*	D	A*	C
Sinkeviciute, 2020 (69)	A*	B	C	B		D	A*	C
Skoumal, 2008 (88)	A*	B	B	A*		D	A*	C
Smith, 2020 (36)	A*	B	A*	A*		D	A*	C
Souto-Carneiro, 2020 (72)	B	B	B	B	B*	D	A*	C
Stoeckmann, 2006	A*	B	C	A*	A*	D	A*	C
Szodoray, 2007 (52)	A*	B	C	A*	AB**	D	A*	C
van Kuijk, 2010 (90)	A*	A*				D		C
Veale, 1993 (51)	A*	B	C	A*	AB**	D	A*	C
Vinci, 2020 (50)	A*	B	B	B		D	A*	C
Wade, 2018 (119)	A*	B	B	B		D	A*	C
Wang N., 2022 (81)	A*	A*	B	A*	A*	D	A*	C
Waszczykowski, 2020 (17)	A*	A*	C	A*	A*	D	A*	C
Waszczykowski, 2021 (18)	A*	B	C	A*	A*	D	A*	C
Wcisło-Dziadecka, 2020 (128)	B	B	C	A*		D	A*	C
Winchester, 2012 (28)	A*	A*	B	A*	B*	D	A*	C
Yuan, 2019 (49)	A*	B	B	B	AB**	D	A*	C
Zhang, 2016 (31)	A*	B	A*	A*	B*	D	A*	C
Zhu, 2021 (77)	A*	A*	B	B	A*	D	A*	C

**Selection.** 1) Is the case definition adequate? A: yes, with independent validation*; B: yes, eg record linkage or based on self-reports; C: no description.

2) Representativeness of the cases A) consecutive or obviously representative series of cases*; B) potential for selection biases or not stated.

3) Selection of Controls A: community controls*; B: hospital controls; C: no description.

4) Definition of Controls A: no history of disease (endpoint)*; B: no description of source.

**Comparability.** Comparability of cases and controls on the basis of the design or analysis A: study controls for …*; B: study controls for any additional factor*.

**Exposure.** 1) Ascertainment of exposure A: secure record (eg surgical records)*; B: structured interview where blind to case/control status; C: interview not blinded to case/control status; D: written self-report or medical record only; E: no description.

2) Same method of ascertainment for cases and controls A: yes*; B: no.

3) Non-Response rate A: Same rate for both groups*; B: non respondents described; C: rate different and no designation.

**Table 4:** Assessment of bias risk using NOS scale for cohort study.

References	Representativeness of the exposed cohort	Selection of the non-exposed cohort	Ascertainment of exposure	Demonstration that outcome of interest was not present at start of the study	Comparability of cohorts based on the design or analysis	Assessment of outcome	Was follow-up long enough for outcomes to occur	Adequacy of follow up of cohorts
Abji F, 2020 (70)	B*		A*	A*		A*	A*	B*
Abji F, 2020 (BMJ) (70)	B*	A*	A*	A*	B*	A*	A*	D
Chandran, 2013 (29)	B*		B*	A*		A*	A*	A*
Fuentelsaz-Romero, 2021 (76)	B*	A*	A*	A*	A*	A*	C	D
Inciarte-Mundo, 2018 (118)	B*	A*	B*	A*	B*	A*	A*	B*
Iwaszko, 2021 (41)	A*	A*	A*	A*	A*	A*	A*	A*
Wade, 2020 (40)	A*	A*	B*	A*	A*	B*	A*	D

1) Representativeness of the exposed cohort A: truly representative of the average in the community*; B: somewhat representative of the average in the community*; C: selected group of users eg nurses, volunteers; D: no description of the derivation of the cohort.

2) Selection of the non-exposed cohort A: drawn from the same community as the exposed cohort* B: drawn from a different source; C: no description of the derivation of the non-exposed cohort.

3) Ascertainment of exposure A: secure record (eg surgical records)*; B: structured interview*; C: written self report; D: no description.

4) Demonstration that outcome of interest was not present at start of study A: yes* B: no.

5) Comparability of cohorts on the basis of the design or analysis A: study controls for … *; B: study controls for any additional factor*.

6) Assessment of outcome A: independent blind assessment*; B: record linkage*; C: self report; D: no description.

7) Was follow-up long enough for outcomes to occur A: yes (select an adequate follow up period for outcome of interest)*; B: no.

8) Adequacy of follow up of cohorts A: complete follow up - all subjects accounted for*; B: subjects lost to follow up unlikely to introduce bias*; C: follow up rate < ::% (select an adequate %) and no description of those lost; D: no statement.

## 4 Discussion

Our systematic review of the literature shows that few biomarkers are currently available to guide clinical practice in the diagnosis and prognosis of PsA. This review has highlighted COMP and MMP3 as two potential serum biomarkers for the diagnosis of PsA. However, the discriminative qualities of these bone and cartilage remodeling markers have been revealed as insufficient for clinical use.

The meta-analysis showed that COMP was able to differentiate patients with PsA from those with OA or from healthy subjects. However, serum levels varied widely between studies. One study reported mean COMP rates were 10-fold lower in the PsA, OA, and HC populations compared with those observed in the other studies, while methodologically, only the commercial ELISA kits differed between studies (15). The success of COMP in distinguishing PsA from PsO was different across studies, and the absence of numerical data prevented performance of a meta-analysis and to deduce its efficacy in this application.

The pooled serum MMP-3 levels were significantly higher in PsA patients than in PsO patients. However, the meta-analysis showed that they did not appropriately distinguish patients with PsA from healthy subjects. Similarly, the only publication that studied MMP3 serum levels in PsA and OA reported no difference between the two (134). MMP3 levels were increased in OA when compared to HC, but do not appear to be a reliable biomarker, particularly as their assessment in a multiplex system reported results contrary to those found in this analysis (i.e., lower MMP3 serum levels in patients with PsA and PsO than in healthy subjects) (16). None of the remaining bone remodeling or cartilage markers demonstrated any ability to differentiate patients with PsA from controls. However, all the studies included in the analysis were cross-sectional studies, with patients whose diagnosis was pre-existing and whose disease duration was not considered.

We have not identified any new genetic biomarkers useful in the diagnosis of PsA since the last meta-analyses on the subject, and these analyses on genetic polymorphisms had not identified any useful biomarkers for the practical diagnosis of PsA (135, 136). Our systematic review did not identify all publications on HLA and PsA association, which may be due to the fact that our search algorithm was not specifically focused on genetics. The major loci of interest have historically been MHC region and HLA genes, of which certain alleles, primarily HLA-C*06 and HLA-B*27, are carried by about 20-35% of PsA patients (137). Recently, HLA-B27 has been identified as a marker of the axial PsA phenotype, and HLA-C*06 as a marker of the peripheral PsA phenotype (138). Furthermore, both systematic review for PsA biomarkers and a recent meta-analysis examining HLA association in PsA patients confirmed a significant increase in the risk of PsA in HLA-C*02 and C*12 populations (139, 140). Specific non-HLA PsA variants have been identified in GWAS studies, including in the IL12B, NOS2 and IFIH1 regions as reported in a systematic review published in 2015 (141). Several signaling pathways possibly implicated in the pathogenesis of PsA were presented in a recent systematic review published in 2020 (142).

Data concerning autoantibodies in PsA remains sparse in the literature. While some data appears promising, no replication studies have been published. Anti-CCP antibodies are associated with polyarticular phenotypes and structural lesions, and have been shown to be markers of severity rather than diagnosis. Indeed, a recent study reported a correlation between anti-CCP antibodies in PsA and pulmonary manifestations (143).

This systematic review of the literature has several limitations. Only patients with PsA were included while studies involving patients with SpA were excluded, which may have incidentally excluded patients with psoriatic forms of axial disease. The search algorithm also did not include imaging biomarkers, although combining imaging with other biomarkers might help to better define PsA (144). In addition, we choose to focus on diagnosis and prognosis biomarkers unrelated to treatment and did not include predictive biomarkers of treatment response. Finally, our search equation did not allow us to highlight the numerous studies concerning genetics biomarkers in psoriatic arthritis either and should be a focus of a future systemic review.

In summary, this review was broad, with more than 50 studies included since the prior systematic review on diagnostic and prognostic biomarkers in PsA (139). No specific diagnostic biomarkers for PsA were identified, despite the fact that this was the first meta-analyses to assess COMP and MMP3. The search for autoantibodies in PsA appears promising but requires additional confirmatory studies. Further studies are also needed to assess the performance of potential biomarkers that can distinguish PsA from OA and other chronic inflammatory diseases.

## Data availability statement

The original contributions presented in the study are included in the article/Supplementary Material, further inquiries can be directed to the corresponding author/s.

## Author contributions

TW: conception and design of the study, acquisition of data, analysis of data, original draft preparation, data curation, reviewing and editing. NB: conception and design of the study, editing LB: analysis of data, stats PL: editing, supervision TP: conception and design of the study, analysis of data, drafting manuscript, supervision, reviewing and editing All authors reviewed and approved the final draft of the manuscript.

## Acknowledgments

Mrs. Catherine Weill assisted with the Embase bibliographic research. JetPub Scientific Communications LLC provided editorial assistance to the authors during preparation of this manuscript.

## Conflict of interest

The authors declare that the research was conducted in the absence of any commercial or financial relationships that could be construed as a potential conflict of interest.

## Publisher’s note

All claims expressed in this article are solely those of the authors and do not necessarily represent those of their affiliated organizations, or those of the publisher, the editors and the reviewers. Any product that may be evaluated in this article, or claim that may be made by its manufacturer, is not guaranteed or endorsed by the publisher.
